# Metabolomics Reveals Metabolic Biomarkers of Crohn's Disease

**DOI:** 10.1371/journal.pone.0006386

**Published:** 2009-07-28

**Authors:** Janet Jansson, Ben Willing, Marianna Lucio, Ages Fekete, Johan Dicksved, Jonas Halfvarson, Curt Tysk, Philippe Schmitt-Kopplin

**Affiliations:** 1 Ecology Department, Division of Earth Sciences, Lawrence Berkeley National Laboratory, Berkeley, California, United States of America; 2 Department of Microbiology, Swedish University of Agricultural Sciences, Uppsala, Sweden; 3 Helmholtz-Zentrum Muenchen-German Research Center for Environmental Health, Institute for Ecological Chemistry, Neuherberg, Germany; 4 Department of Medicine, Division of Gastroenterology, Örebro University Hospital and School of Health and Medical Sciences, Örebro University, Örebro, Sweden; Charité-Universitätsmedizin Berlin, Germany

## Abstract

The causes and etiology of Crohn's disease (CD) are currently unknown although both host genetics and environmental factors play a role. Here we used non-targeted metabolic profiling to determine the contribution of metabolites produced by the gut microbiota towards disease status of the host. Ion Cyclotron Resonance Fourier Transform Mass Spectrometry (ICR-FT/MS) was used to discern the masses of thousands of metabolites in fecal samples collected from 17 identical twin pairs, including healthy individuals and those with CD. Pathways with differentiating metabolites included those involved in the metabolism and or synthesis of amino acids, fatty acids, bile acids and arachidonic acid. Several metabolites were positively or negatively correlated to the disease phenotype and to specific microbes previously characterized in the same samples. Our data reveal novel differentiating metabolites for CD that may provide diagnostic biomarkers and/or monitoring tools as well as insight into potential targets for disease therapy and prevention.

## Introduction

Crohn's disease (CD) is an inflammatory bowel disease (IBD), characterized by chronic inflammation of the gastrointestinal tract. The exact etiology of CD is unknown, but both the host genotype and environmental factors play a role, and it is known that disease induction requires the presence of bacteria. No specific pathogen has been defined as a causative agent, but individuals with CD have an imbalance or ‘dysbiosis’ of their intestinal microbiota, or microbiome [1], [2]. Dysbiosis in turn leads to a breakdown in the détente relationship between the microbiome and the host immune system, through unknown mechanisms.

Diagnostic and monitoring tools for CD are currently inadequate. Numerous serological biomarkers have been proposed for the diagnosis of CD. However, for clinical applications, none of the current markers stand-alone and they are therefore used in conjunction and as a supplement to endoscopy. Therefore, more accurate tools are needed for early diagnosis of CD; in particular non-invasive approaches that can be used in place of endoscopy.

Our aim was to search for metabolic biomarkers of CD as evidence of microbial functions in the gut. Recent advances in nuclear magnetic resonance (NMR) and mass spectrometry (MS) have made it possible to simultaneously assess thousands of metabolites corresponding to the “metabolome” and to determine end-points of metabolic processes in living systems [3]. NMR (^1^H NMR Spectroscopy) has revealed gut microbial contributions to metabolite compositions in different body fluids, including blood [4], urine [5], [6] and fecal extracts [7], [8]; and the latter has revealed some metabolites that are correlated to CD [7].

Although the information provided using NMR has been very valuable it is still limited by low resolution and sensitivity that only enables the annotation and quantification of a limited number of lower molecular weight molecules. By contrast, ion cyclotron resonance-Fourier transform mass spectrometry (ICR-FT/MS) with an ultrahigh mass resolution enables differentiation of very subtle variations in thousands of mass signals, including higher molecular weight metabolites [9]. The combination of coupled metabolite separation technologies to spectrometry and spectroscopy enables a multidimensional approach for the structural identification of new metabolites as recently exemplified for markers of diabetes and early stage insulin resistance [10]. Therefore, in this study we used ICR-FT/MS with its high dynamic range and high mass accuracy (0.2 ppm) to obtain non-targeted profiles of elementary compositions in fecal samples obtained from individuals with CD.

## Analysis

### Patient cohort

The selection criteria and patient information for the twins studied in this experiment have previously been described [11], [12]. Patient information presented in Table S1, including responses to a questionnaire regarding the usage of antibiotics, non-steroidal anti-inflammatory drugs during the preceding 12 months, gastroenteritis within the preceding 3 months, and specific dietary habits have previously been reported [12]. Written informed consent was obtained and approved by the Swedish ethical committee. The sample cohort was comprised of 15 twin pairs, with 7 healthy twin pairs, 4 pairs that were discordant for predominantly colonic CD (CCD), 2 pairs that were discordant for predominantly ileal CD (ICD), 2 pairs that were concordant for ICD, and 2 pairs that were concordant for CCD. There were a total of 8 individuals with CCD (aged 20–70, mean 48), 6 individuals with ICD (aged 44–53, mean 49.8), 6 healthy individuals with a sick twin (HD) (ages 20–70, mean 51.8) and 14 healthy individuals with a healthy twin (HH). In the HH group, 5 pairs were children (ages 5–11, mean 7.4) and 2 pairs were of similar ages to diseased individuals (45–55 mean 50). All diseased individuals were in clinical remission according to the Harvey-Bradshaw score [13] with the exception of 2 individuals (10b and 15a). Both of these individuals had endoscopic recurrence scores below 2 [14] at colonoscopy suggesting that the Harvey-Bradshaw score did not indicate active CD. Fecal samples were placed in a freezer at −70 °C within 24 h of collection, until analysis. The use of human subjects for this study was approved by the Örebro County Ethical Committee (Dnr; 167/03).

### Preparation of fecal water

Fecal samples were diluted 1∶60 (weight∶volume) in cold (4°C) 50 mM sodium phosphate buffer (pH 8.0). The suspension was mixed in a gyratory shaker at 120 rpm for 10 min at 4°C and then centrifuged at 200 x g for 10 min at 4°C to pellet the debris. The supernatant was removed and centrifuged at 18,000 x g for 10 min to pellet the bacteria. The supernatant was collected and the pH was lowered to approximately 4.5 by addition of 0.25 mL of 1% formic acid to each mL of supernatant, with a resulting final sample dilution of 1∶75. The fecal water samples were frozen and stored frozen at −70°C until analysis. The samples were extracted 20 min after they were thawed at room temperature by solid phase extraction (SPE) using 1 L cartridges filled with 100 mg of octadecy-bonded silica packing (Bakerbond, Mallinckrodt Baker, Griesheim, Germany) for desalination and deproteination of the sample. The cartridges were preconditioned with 2 mL of methanol and 2 mL of water acidified with 0.1% formic acid prior to the application of 0.5 mL of sample. The columns were washed with 0.5 mL of 0.1% formic acid and metabolites were eluted with 0.5 mL methanol.

### FT-ICR-MS analysis

High-resolution mass spectra (resolution Δ(m/z)/(m/z) of 500.000 at m/z 500 in full scan mode) were acquired on a Fourier Transform Ion Cyclotron Resonance Mass Spectrometer (Bruker, Bremen, Germany), equipped with a 12 Tesla superconducting magnet and an Apollo II ESI source. Samples were infused with the micro-electrospray source at a flow rate of 120 µL/h with a nebulizer gas pressure of 20 psi, and a drying gas pressure of 15 psi at 250°C. Negative and positive electrospray ionisation was used. Spectra were externally calibrated on clusters of arginine (m/z of 173.10440, 347.21607, 521.32775 and 695,43943) dissolved in methanol at a concentration of 10 mg/L; calibration errors in the relevant mass range were always below 0.1 ppm. Four MW time domains were applied in the mass range of 150–2000 m/z. The ion accumulation time in the ion source was set to 2 s and 200 scans were accumulated per sample. Before Fourier transformation of the time-domain transient, a sine apodization was performed. The raw data were processed with DataAnalysis 3.4 (Bruker Daltonik, Bremen) software that is hard-coded in the instrument. Peaks exceeding a threshold signal-to-noise ratio of 3 were exported to peak lists. The extracted spectra were aligned though in-house software.

### Identification of differentiating masses and assignment to metabolic pathways

Different multivariate analysis techniques including principal component analysis (PCA), hierarchical cluster analysis (HCA) and partial least square discriminant analysis (PLS-DA) were combined to reduce the data sets into a series of optimized and interpretable objects. The study of contribution of the different variables (m/z in this case) was done though the analysis of the regression coefficients. The statistical analyses were done with SIMCA-P 11.5 (Umetrics, Umea, Sweden), SAS version 9.1 (SAS Institute Inc., Cary, NC, USA) and the Statistical package “R” (v 1.8.1) for the heatmap visualization.

The masses with the highest regression coefficient (an arbitrary cut off value >0.0004) were chosen to be discriminant. Moreover these masses have a variable importance in the projection VIP>1 (where a VIP value of ≥1 is regarded as significant). The VIP is a computation of the influence of every x term in the model on the y variable (ICD, CCD, and Healthy). Larger VIP values indicate a greater influence of a term x on the y variable.

We used a PLS-DA model to evaluate the m/z that contributed to separation between healthy vs individuals with CCD and ICD. The model gave good values for Q^2^(cum) = 0.6 and R^2^(Y) = 0.9. However the permutation test applied with 100 permutations revealed a possible over fitting of the model. Thus the dataset was reduced excluding the masses with frequencies less than three and a PLS model with an orthogonal signal correction (OSC) was applied. The putative masses responsible for the metabolic differentiation were used to make queries in the KEGG database (Kyoto Encyclopedia of Genes and Genomes) through the MassTRIX software [15] including *Homo sapiens* and *Bacteroides vulgatus* as reference species. MassTRIX calls the KEGG/API (http://www.genome.jp/kegg/soap/) to generate pathway maps, where the annotated compounds and genes are highlighted using different colors-thus differentiating between organism-specific and extra-organism items [15]. The identification of certain metabolites as their exact masses in their given biological context was strategic in the context of searching for biomarkers for CD.

### Correlation of metabolites to microbial profiles

The bacterial community profiles of the same fecal samples studied here have previously been reported [12]. To determine correlation between the microbial community composition and the metabolic profiles, distance matrices using Manhattan distances [16] for microbial and metabolic profiles were calculated independently, then Pearson correlation coefficients between the two distance matrices were calculated. Significances of the correlations were tested using the Mantel test with 1000 permutations. Cluster analysis of microbial and metabolic profiles was performed using binary data (Jaccard's similarity index). Moreover the relation between the metabolites and the microbial profiles was analyzed with a PLS model with OSC, the metabolites were used as explicative variables. The relation of the first ten explicative masses with the different bacteria was expressed with the heatmap.

## Discussion

Due to the tremendous individual diversity in the composition of the gut microbiota in humans and the corresponding anticipated diversity in the metabolites they produce, we expected it to be challenging to correlate specific metabolites to CD. Therefore, in this study, we focused on a twin cohort, described previously [2], [17], that includes healthy twin pairs, concordant pairs (both twins have CD) and discordant pairs (one twin is healthy and the matched twin has CD). The patients with CD were further differentiated depending on whether inflammation was primarily localized in the ileum (ICD) or in the colon (CCD) [2], [17]. A particular value of this patient cohort was the availability of existing data about the microbial profiles in fecal samples [17] and biopsies [2] from the same individuals that enabled the possibility of correlation of metabolites to microbial populations. Our previous studies showed that there were significant differences in levels of several members of the microbial communities in the gut of individuals that had ICD compared to those with CCD or to healthy individuals; in particular dramatically lower abundances of *Faecalibacterium prasnitzii* and higher levels of *Escherichia coli* in individuals with ICD compared to the other two groups [2]. However, there was no clear distinction between the microbial community profiles in healthy individuals and those with CCD. Although the gut microbial communities of healthy twins were more similar to each other than to other individuals in the sample cohort, this similarity was no longer evident when comparing twin sets where one or both twins were sick [17].

In this study, we used several multivariate statistical approaches to analyze the metabolites present in the liquid phase (fecal water) of the same fecal samples examined earlier [17]. First, using principal components analysis (PCA) we found that the metabolomes of individuals that had ICD grouped separately from those with CCD and from healthy individuals (Figure S1). Some of the healthy individuals were young (Table S1) and their metabolomes grouped separately from the healthy adults on the PCA plots (Figure S1). This distinction between healthy young and old was not evident in our previous analyses of the microbial community compositions [17]. An example of a discriminating metabolite that contributed highly to the grouping of the young was 5β-cyprinosulfate, a bile acid that was more abundant in young subjects (*P*<0.003) compared to all other groups. Because there were no young individuals with CD in this study, we continued with adults only for further statistical discrimination of diseased from healthy groups.

**Figure 1:**
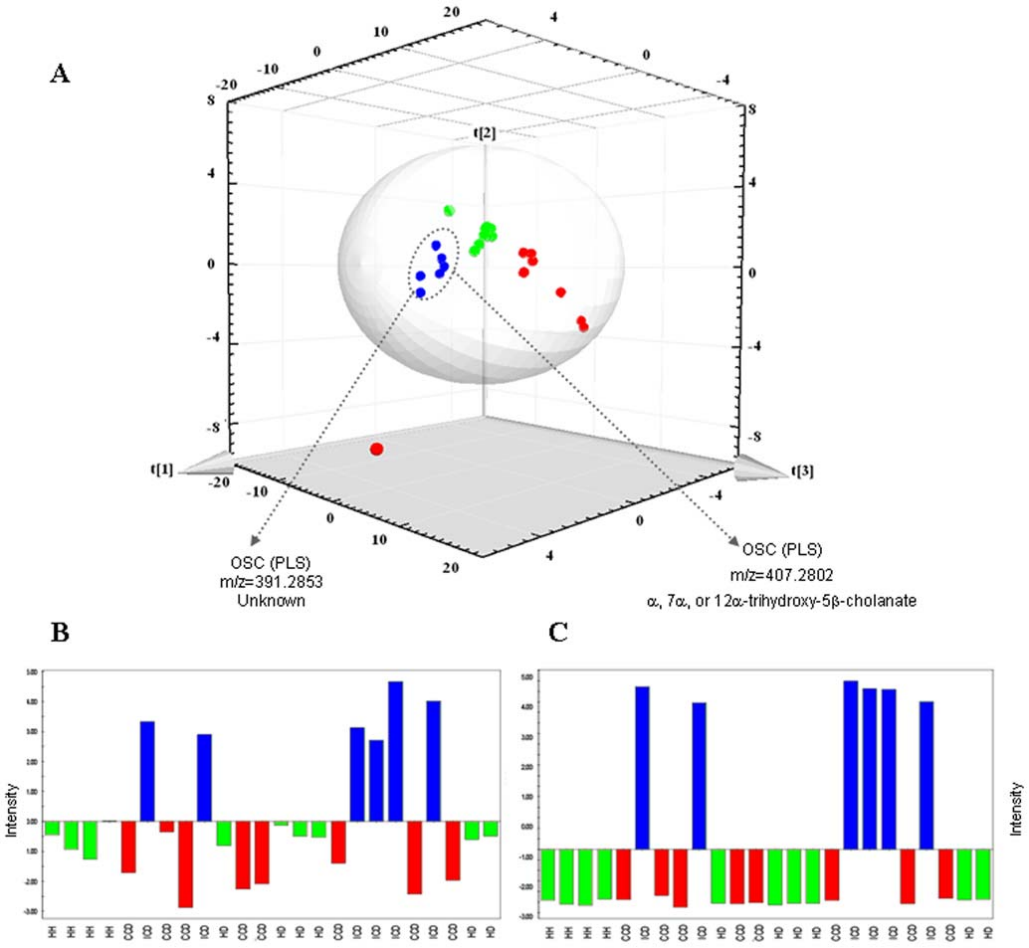
(A) Score and loading scatter plot of PLS analysis (Q^2^(cum) = 0.96, R^2^(Y) = 0.95). (blue • = ICD, red • = CCD and green • = Healthy). The masses with the highest regression coefficients were considered as discriminant. Coordinates on the figure axes are ×10^8^. (B) Example of a differentiating metabolite for ICD (assigned at m/z of 391.2853) that is up regulated in the ICD group but the structure is unknown. (C) Mass at m/z of 407.2802 corresponding to 3α, 7α, or 12α-trihydroxy-5β-cholanate within the bile acid biosynthesis pathway. The intensities in B and C were normalized.

Using a partial least squares (PLS) statistical approach on corrected mass data the separation between disease phenotypes was even more pronounced than when using the PCA model, with a clear separation of individuals with ICD from those with CCD and from healthy individuals (Fig. 1A) and some examples of differentiating metabolites are shown in Figure 1B. This differentiation according to disease phenotype that was seen using both the PCA and PLS approaches provides further support to the recent hypothesis that ICD and CCD are different disease phenotypes of CD. The outlier with CCD was the youngest of our Crohn's patients (born 1986) and had only had the disease for 4 years at the time of sampling, whereas all the others have had the disease for >10 years.

The 2^nd^ component of the PLS model also revealed a clear separation, not only between the individuals with ICD versus CCD and healthy, but also between CCD and healthy. These data are the first that we have seen from this sample cohort that differentiate healthy from CCD individuals. Therefore, the resolution of separation of the groups was higher for the metabolite profiles than for the microbial community profiles [17], thus demonstrating the potential for use of metabolomics as an approach for accurate disease diagnosis. One reason for the higher discrimination of the metabolite data compared to the microbial data could be the direct link of metabolites to function since they represent the final signature of enzymatic processes occurring in the gut. By comparison, detection of microbial presence based on DNA targets may be misleading since many species in the gut may be dormant, dead, or transient and this information is not available when assessing DNA alone. Another explanation for the difference between DNA-based surveys and metabolite-based surveys could be that some microbes may be present and active in both diseased and healthy individuals, but may not have a significant effect on levels of the metabolites that we are screening for.

**Figure 2:**
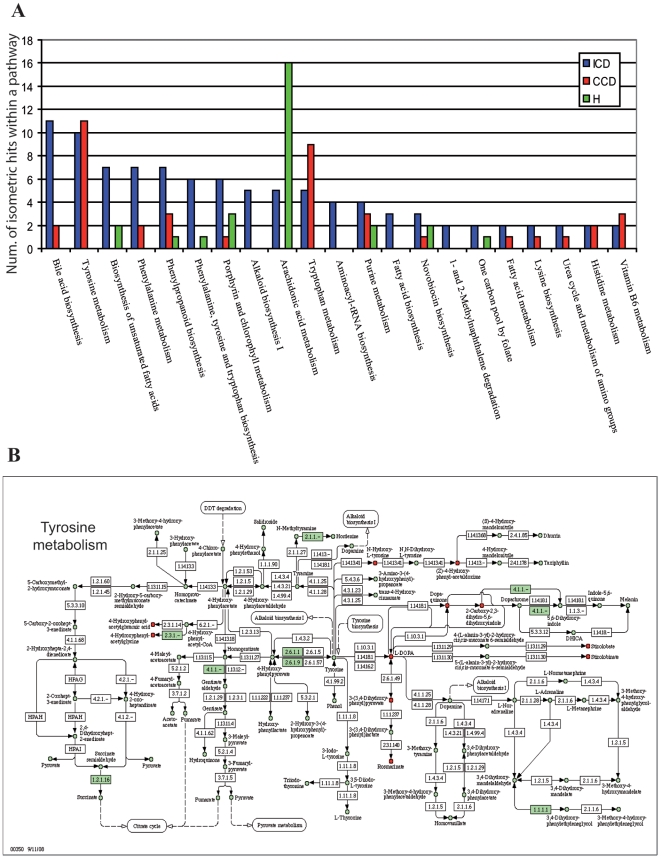
(A) KEGG pathways that discriminated the three groups: ICD CCD and healthy. The m/z were selected after the validation of PLS model; (B) Tyrosine metabolism pathway, the red metabolites were identified and present in the ICD group. Green shading refers to enzymes that were annotated in *Bacteroides vulgatus*.

Metabolites within a broad range of pathways contributed to the differentiation of healthy from diseased individuals, as well as between disease phenotypes (Fig. 2A, Table 1). From the total number of 18,706 measured masses, we found that 7919 were discriminating for a specific disease phenotype: 2155 for ICD, 3113 for CCD and 2650 for healthy.

**Table 1:** Identified fecal metabolites that contribute to the discrimination of disease phenotypes.

Pathway	Metabolite	CCD		Healthy		ICD		
		Mean^1^	Detected (n/8)	Mean^1^	Detected (n/10)	Mean^1^	Detected (n/6)	*P*
Tyrosine metabolism	2-Carboxy-2,3-dihydro-5,6-dihydroxyindole/dopaquinone	202889263_a_ *	5	133363_ b_	1	2616539_ b_	4	.015
	4-hydroxyphenyl-acetylglycine	8699313_ a_ *	5	0_ b_	0	140068_ b_	1	.012
	(Z)/4/hydroxyphenyl-acetaldehyde-oxime	502918_ a_ *	4	0_ b_	0	0_ b_	0	.006
Amino acids	Tyrosine	335531_ b_	3	86251_ b_	1	1441491_ a_ *	6	.001
	Tryptophan	208343_ b_	2	0	0	1087303_ a_ *	5	.001
	Phenylalanine	0_ b_	0	0	0	1405997_ a_ *	5	.001
Bile acid metabolism	Glycocholate	715048_ b_ *	4	0	0	1847470_ a_ *	5	.001
	Taurocholate	312872_ b_	2	179560_ b_	2	14227172_ a_ *	5	.002
	Trihydroxy-6β-cholanate	20481784_ b_	8	16410877_ b_	10	1016307115_ a_ *	6	.001
	Chenodeoxyglycocholate/Glycochenodeoxycholate	335746_ b_	2	83591_ b_	1	1200688_ a_ *	5	.002
Fatty acid biosynthesis	Oleic acid	451036_ b_	2	580092_ b_	3	5745324_ a_ *	5	.010
	Stearic acid	661070_ b_	3	687268_ b_	5	2658962_ a_ *	5	.021
	Palmitic acid	357430_ b_	2	184491_ b_	2	2476141_ a_ *	5	.006
	arachidonic acid	0_ b_	0	0_ b_	0	715296_ a_ *	3	.004
	octadecatrienoic acid	0_ b_	0	0_ b_	0	568143_ a_ *	3	.005
	linoleic acid	920810_ b_	3	778285_ b_	4	5202328_ a_ *	5	.022
Arachidonic acid	prostaglandin F2α	607077	4	1400944*	9	977053	4	.142
metabolism/prostaglandins	2,3-dinor-8-iso-prostaglandin F2α	4002093	8	4048724*	10	1918427	6	.093
	prostaglandin F1α	1216394_ b_	7	2482457_a_ *	10	812282_ b_	4	.017
	prostaglandin E2α	1726834	8	2662085*	9	1077826	5	.088
Phenylalanine metabolism	3-(4-hydroxy-phenyl)propionic acid/3-(4-hydroxyphenyl)lactate	2945178_ ab_ *	4	513000_ b_	4	3632083_ a_ *	6	.001

* Indicates the group discriminated by the given metabolite in PLS-DA; n, indicates number out of the total number of individuals in each disease category.

1 Mean, refers to the mean amount (daltons) of the metabolite detected in all individuals within a given disease category.

P values indicate a significant difference between groups based on Anova; different subscript letters indicate groups that differed significantly (<.05).

Of the discriminating masses 13.3%, 9.5% and 9.3% for ICD, CCD and healthy groups, respectively, could be assigned to metabolic pathways using MassTRIX. However, 89.6% could not be assigned using MassTRIX software indicating that the available databases used for the identification of masses are yet limited by incompleteness. Some of the unassigned masses were interesting, such as the negative ion at mass-to-charge ration (m/z) of 229.1557 or 391.2853 (see Fig. 1B), that were characteristic for ICD patients. Pathways with differentiating metabolites included those involved in the metabolism and or synthesis of amino acids, fatty acids, bile acids and arachidonic acid and these are discussed in more detail below.

We found numerous masses corresponding to metabolites within the tyrosine metabolic pathway (Fig. 2B) that discriminated diseased from healthy groups. For example, dopaquinone, an oxidation product of dopa and an intermediate in the formation of melanin from tyrosine, was more abundant (*P*<0.05) in CD patients (both CCD and ICD) than in healthy individuals. L-DOPA has been observed at elevated levels in inflamed mucosa of IBD patients [18]. There is also some evidence that polymorphisms in the dopamine receptor D-2 play a role in CD [19]. In addition, 4-hydroxyphenylacetylglycine and (Z)-4-hydroxyphenylacetaldehyde-oxime contributed substantially to the separation of CCD individuals. In a previous study 4-hydroxyphenylacetylglycine in urine was negatively correlated to the abundance of Faecalibacterium prausnitzii in the gut [5]. We observed the same negative correlation for healthyIndividuals, but in contrast we found higher levels of 4-hydroxyphenylacetylglycine levels in the feces of a subset of CCD patients with elevated F. Prausnitzii abundances previously published from the same samples [2]. These differences could be due to the different sample origins in the different studies; i.e. urine compared to feces. In our previous studies and others F. prausnitzii was more abundant in healthy individuals and those with CCD compared to individuals with ICD [2], [20]. These results suggest there could be a link between *F. prausnitzii* and the metabolite 4-hydroxyphenylacetylglycine that warrants further study.

Increased amounts of metabolites involved in tyrosine metabolism coincides with earlier reports of increased transcripts of genes involved in tyrosine metabolism in peripheral blood mononuclear cells from CD patients [21]. However, it is not clear what (if any) role increased tyrosine metabolism may play in CD. Interestingly, protein tyrosine phosphatases have been associated with autoimmune diseases [22], although such mutations have not been correlated to CD.

The amino acids tryptophan and phenylalanine were also indicative of the ICD phenotype (Table 1). The presence of tryptophan in the feces might correspond to a subset of previously described CD patients with a specific depression of blood tryptophan levels [23]. Interestingly, one ICD individual did not have detectable levels of tryptophan or phenylalanine, and this individual also had the lowest abundance of mucosal *E. coli* [2], compared to the other ICD individuals. Although other organisms could also be correlated to these metabolites, it would be of interest to further investigate any links between *E. coli* abundance and amino acids in the gut. Previous studies [24] have shown that *E. coli* isolated from CD patients have pathogen-like behavior in vitro, and may play a role in the inflammatory process. Marchesi et al. [7] also observed increased levels of some other amino acids in fecal samples of CD patients with active disease (ours were in remission), but these were different amino acids than those we found; i.e. alanine, isolueucine, leucine and lysine. The presence of amino acids in the feces of ICD patients in general may be the result of malabsorption resulting from the shortening of the small bowel or due to subclinical inflammation, or conversely the result of secretion into the bowel. Regardless of the underlying mechanism, these results suggest that a special consideration of amino acid balance should be made for patients with active disease as well as those in remission.

Many masses corresponding to metabolites within the bile acid biosynthesis pathway contributed to the segregation of disease phenotypes. In particular, the mass corresponding to glycocholate was prevalent in a majority of individuals with CD (Table 1), but not detected in healthy individuals. Masses corresponding to taurocholate, 3α, 7α, 12α-trihydroxy-5β-cholanate and chenodeoxyglycocholate were also particularly high (*P<*0.001) in ICD patients (Table 1). The majority of bile acids are reabsorbed in the distal ileum, largely accomplished by an apical sodium dependent bile acid transporter, and thus ileal resection could explain the reduced bile acid absorption in ICD patients. However, only 1 of the 4 CCD patients with glycocholate in the feces had undergone resection surgery. This is consistent with the finding that patients with and without ileal resection have altered bile composition [25]. Increased bile in the feces may indicate that although these subjects were in remission they were nevertheless experiencing sub-clinical inflammation as bile acid absorption has been shown to be inhibited by inflammation [26], or increased mucosal permeability [27]. A detrimental feedback loop could be created where inflammation results in reduced bile absorption, therefore increased bile in the lumen, which in turn causes increased inflammation.

Masses corresponding to both saturated and unsaturated fatty acids, including oleic acid, stearic acid, palmitic acid, 6Z-, 9Z-, and 12Z-octadecatrienoic acid, linoleic acid and arachadonic acid, were also higher in patients with ICD compared to the other groups (Table 1). Fernandez Baneres *et al*. [28] previously reported elevated levels of arachidonic acid and linoleic acid in colonic mucosa of CD patients, consistent with our results from fecal samples, but they also found reduced amounts of oleic acid in the mucosa, contrary to the increased levels we observed.

Arachidonic acid is particularly interesting because it is known to mediate inflammation and the functioning of several organs and systems either directly or upon its conversion into eicosanoids. Arachidonic acid has previously been shown to increase the expression of the intracellular adhesion molecule (ICAM)-1, which is involved in the recruitment of leukocytes, suggesting another role of this molecule [29]. Linoleic acid and arachidonic acid are also essential for the synthesis of prostaglandins (PG), which are important immune signaling molecules. However, in our study the increased abundances of these fatty acids did not correspond to levels of PG (see below) and further studies are necessary to determine their possible links and correlations to CD.

Masses corresponding to PG and their breakdown products including PGF2α and 2,3-dinor-8-iso-PGF2α, thromboxane/6-Keto-PGF1α/PGI2 and PGE2 were more prevalent in healthy individuals (including the young group) than those with CCD and ICD (Table 1). The role of PG as immune signaling molecules is particularly interesting as CD is associated with disregulated immune function. CD-associated alleles have recently been negatively correlated to quantitative expression levels of prostaglandin receptor EP4 (PTGER4) [30] and PTGER4 knockout mice experience more severe colitis in the dextran sodium sulfate model of colitis. The reduced levels of PGs that we observed may reflect a reduced absorption of their precursors, arachidonic acid and linoleic acid, as indicated above. While we observed reduced prostaglandin levels in CD patients that were in remission PG particularly PGE2, have been observed to be more abundant in patients with active CD [31]. This is consistent with the fact that PGE2 is proinflammatory, acting through the EP-2/4 receptor on dendritic cells inducing the expression of IL-23 resulting in a TH17 phenotype associated with CD [31]. Therefore, reduced PG levels in CD patients in remission observed here may make them susceptible to relapse, although elevated levels during active disease could be a cause of tissue damage. Intriguingly, 3-(4-hydroxyphenyl)-propionic acid, that has previously been shown to suppress PGE2 production [32], was particularly elevated in a subset of CCD and ICD individuals.

**Figure 3:**
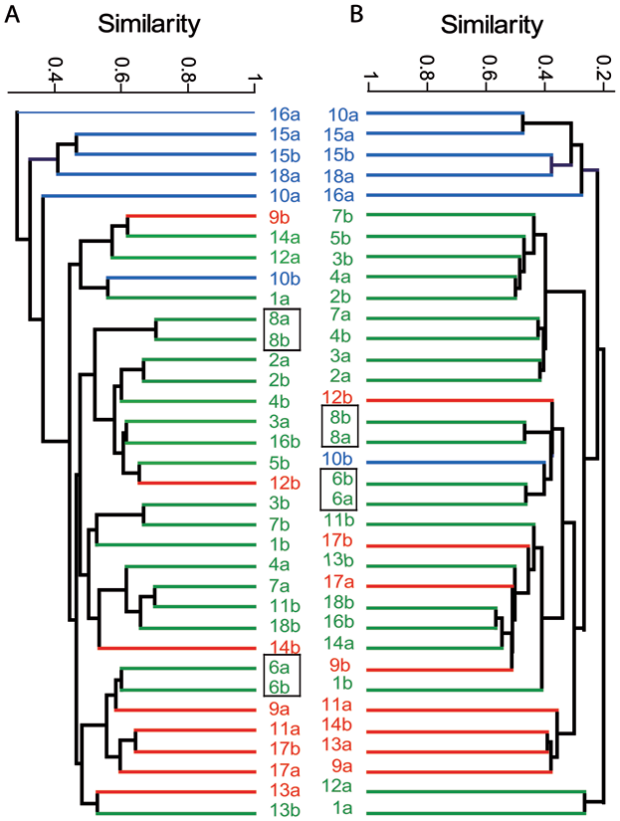
Similarity plot (using Jaccard's index) of (A) microbial composition based on binary T-RFLP data and (B) ICR-FT/MS data, respectively, from fecal samples of individuals with ICD (blue), CCD (red) and healthy individuals (green). Individuals were numbered according to Table S1, and as previously defined (*11*). Boxes indicate twin pairs that share the most similar metabolic and microbial profiles. Note: metaproteome data from the same fecal samples for individuals 6a and 6b have recently been published [33].

Manhattan distances were calculated from the metabolic profiles of all individuals to determine whether the gut metabolomes of twins were more similar to each other than to unrelated individuals. The inter-twin similarity (mean±SE) of healthy twins (0.513±0.035) and concordant twins (0.431±0.070) was greater (*P*<0.001) than that of discordant twins (0.276±0.048). This coincides with the reduced similarity of microbial profiles previously observed in the discordant twins [17]. A correlation between the metabolic and microbial distance matrices (r = 0.348, *P<*0.001) coincides with findings from Li et al. [5] correlating fecal microbial profiles to urinary metabolites, indicating a contribution of bacteria to overall metabolic profiles in the human host. We observed a stronger correlation between metabolic and microbial similarities when making within twin comparisons (r = 0.748, *P*<0.001) strengthening the hypothesis that genetics plays a role in the formation and maintenance of the intestinal microbiome (Fig. 3). The most striking observation from the cluster analysis (Fig. 3) was the similar division of clusters according to the disease phenotype for both the microbial and metabolite data reinforcing the link between microbial community structure, function and disease.

**Figure 4:**
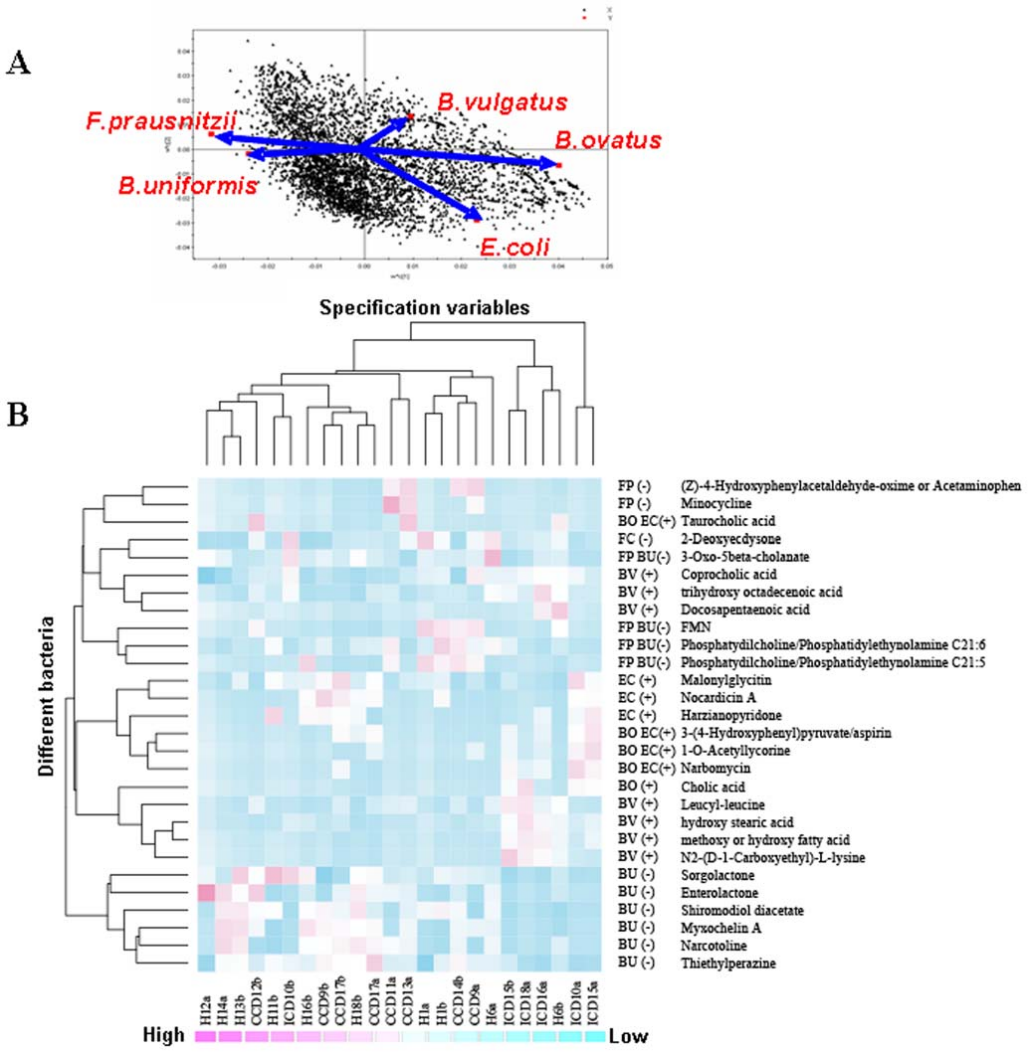
PLS loading plot (A) where bacterial abundance defined the Y matrix and ICR-FT/MS data were plotted as predictors of differentiating bacteria based on their regression coefficients. Masses with the greatest regression coefficients for specific bacterial populations that were more abundant [*B. ovatus* (BO), *B. vulgatus* (BV), and *Escherichia coli* (EC)] and less abundant [*Faecalibacterium prausnitzii* (FP) and *Bacteroides uniformis* (BU)] in the feces of individuals with ileal Crohn's disease (ICD) compared to individuals with colonic Crohn's disease (CCD) and healthy (H) individuals are identified in the heat plot (B). The heat plot indicates the abundance of masses, the predicted metabolite, the bacteria that were positively correlated to that metabolite and whether the metabolite was positively (+) or negatively (−) associated with ICD. The clustering on the x-axis is according to disease and that on the y-axis is according to the relative abundances of the same bacterial populations selected in (A) and corresponding abbreviations are given on the first column to the right of the heat plot. Individuals on the x-axis are coded according to [17]. Each cell is colored based on the detected level of the predicted metabolite.

Subsequently, we also correlated the metabolomes to the relative abundances of specific bacterial populations within the same samples. We used a PLS model with relative abundances of specific microbial populations as the Y matrix and the m/z data as the independent variables to correlate the MS data to microbes and disease status (Fig. 4A). The masses were correlated to predefined key species that we previously found to be significantly more or less abundant depending on the disease phenotype of the host [2], [17]. For example, *Bacteroides vulgatus* (BV), *B. ovatus* (BO) and *E. coli* (EC) were present at significantly higher levels in the ICD group, whereas *F. prausnitzii* (FP) and *B. uniformis* (BU) were more abundant in the healthy and the CCD groups. The 10 masses with the highest regression coefficient value for each of the 5 bacteria indicated above were assigned in MassTRIX (Table S2). The correlation coefficients were computed using bacterial and MS abundances and these data were expressed in a heat map for visualization of the data clustering (Fig. 4B). In this analysis the individuals clustered mainly into two groups: 1) ICD and 2) healthy + CCD, similar to what we found with the PCA plot of the original metabolite data (Figure S1). Bacteria that were more abundant in individuals with ICD (BV, BO and EC) were those that were most strongly correlated to bile acids, including taurocholic and cholic acids, and fatty acids, including stearic, and docosapentanoic acids. Conversely, bacteria that were more abundant in healthy or CD phenotypes (BU and FP) were correlated to phospholipids and flavin mononucleotide (FMN). The correlation of these metabolites to specific bacterial groups merits further attention, such as the causality of the relationship between *E. coli* and elevated levels of taurocholic acid. It should be kept in mind, however, that other bacteria that weren't specifically included in this screening could be contributing to the metabolite profiles seen.

In summary, this study demonstrates the potential of metabolomics to provide a means to differentiate disease phenotypes and to give new insights into the etiology of Crohn's disease. Our study also emphasizes the importance of metabolites produced by the gut microbiota for a healthy gut environment. The analysis procedure was rapid to perform and resulted in highly accurate mass data. Several masses were found that differentiated healthy, ICD and CCD individuals. Interestingly, the similarity of metabolite profiles in healthy monozygotic twin pairs underscores the importance of genetics in determining the nature of the gut environment, including the bacterial species that are most dominant and the metabolites they produce. Further investigation of those masses that were important in differentiation of disease phenotypes, but that could not be assigned to structures, will be an aim for the future using structure identification tools involving hyphenated multidimensional separation, spectrometric and spectroscopic tools.

Our results pinpoint significant differences in the types and number of metabolites within specific pathways, including tyrosine and phenylalanine metabolism and bile acid and fatty acid biosynthesis that could be of key importance for different Crohn's Disease etiologies. Thus, not only the identified individual metabolites, but also the pathways they belong to, could lead to future therapeutic biomarkers or drug targets.

## Supporting Information

Figure S1 Principal component analysis (PCA) scatter plot of fecal metabolites from healthy adults (green filled squares), healthy young (green filled circles), individuals with ileal Crohn's disease (blue), or colonic Crohn's disease (red). The total variance absorbed was 23% and the Eigen values are 6.7 for the first component and 3.38 for the second component.
(0.54 MB TIF)

Table S1 Clinical data for twins and responses to a questionnaire
(0.20 MB DOC)

Table S2 Masses defined through the analysis presented in 5a) and assigned in MassTRIX
(0.11 MB DOC)
