# Supplementary figures and images for: Metabolomics Reveals Metabolic Biomarkers of Crohn's Disease
Source: PLoS One. 2009 Jul 28;4(7):e6386. doi: 10.1371/journal.pone.0006386 (PMC2713417; doi:10.1371/journal.pone.0006386)

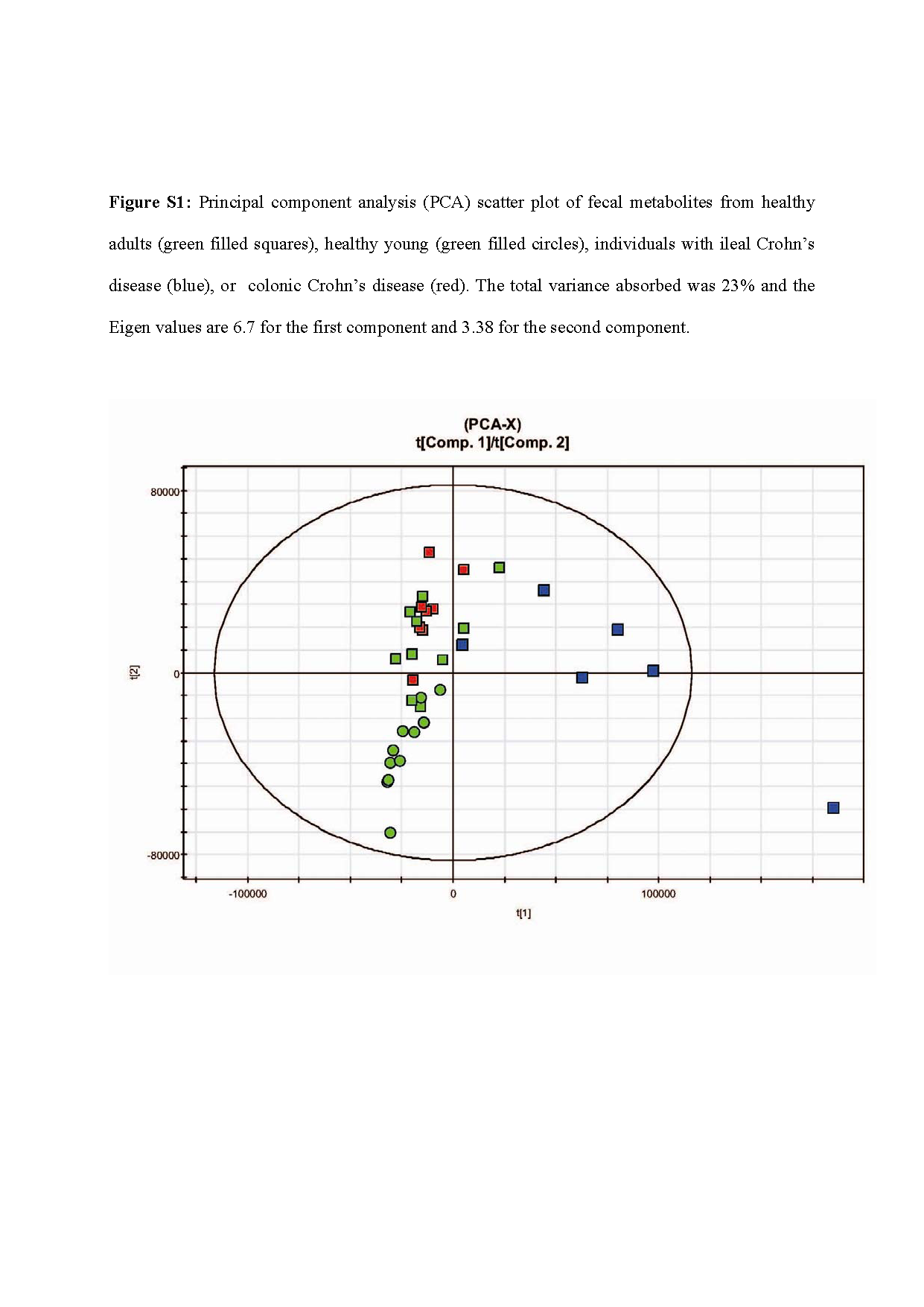

Supplement: Figure S1 — Principal component analysis (PCA) scatter plot of fecal metabolites from healthy adults (green filled squares), healthy young (green filled circles), individuals with ileal Crohn's disease (blue), or colonic Crohn's disease (red). The total variance absorbed was 23% and the Eigen values are 6.7 for the first component and 3.38 for the second component. (0.54 MB TIF) [file pone.0006386.s001.tif]
