# Supplementary material for: Metabolomics Reveals Metabolic Biomarkers of Crohn's Disease
Source: PLoS One. 2009 Jul 28;4(7):e6386. doi: 10.1371/journal.pone.0006386 (PMC2713417; doi:10.1371/journal.pone.0006386)
Supplement: Table S1 — Clinical data for twins and responses to a questionnaire (0.20 MB DOC) [file pone.0006386.s002.doc]

| **Table S1.** Clinical data for twins and responses to a questionnaire. | | | | | | | | | | |
| --- | --- | --- | --- | --- | --- | --- | --- | --- | --- | --- |
| ID | Zygo-city | Status | Birth year | Sex | Nod2 status | Gastro-enteritis | Disease duration | Anti-biotics | NSAID | Surgery |
| 1a | MZ | HH | 1961 | F | nd | No | na | No | Yes | No |
| 1b | MZ | HH | 1961 | F | nd | No | na | No | No | No |
| 2a | MZ | HH | 1995 | M | nd | No | na | No | No | No |
| 2b | MZ | HH | 1995 | M | nd | No | na | No | No | No |
| 3a | DZ | HH | 1999 | F | nd | Yes | na | No | No | No |
| 3b | DZ | HH | 1999 | M | nd | No | na | No | No | No |
| 4a | MZ | HH | 2001 | M | nd | No | na | No | No | No |
| 4b | MZ | HH | 2001 | M | nd | No | na | No | No | No |
| 6a | MZ | HH | 1951 | F | nd | Yes | na | No | No | No |
| 6b | MZ | HH | 1951 | F | nd | No | na | No | Yes | No |
| 7a | MZ | HH | 2000 | F | nd | No | na | No | No | No |
| 7b | MZ | HH | 2000 | F | nd | No | na | No | No | No |
| 8a | DZ | HH | 1998 | M | nd | No | na | No | No | No |
| 8b | DZ | HH | 1998 | M | nd | No | na | No | No | No |
| 9a | MZ | CCD | 1947 | M | wt | No | 19 | No | Yes | No |
| 9b | MZ | CCD | 1947 | M | wt | No | 20 | No | No | No |
| 10a | MZ | ICD | 1962 | F | wt | Yes | 22 | No | Yes | ileal res + right hemi |
| 10b | MZ | ICD | 1962 | F | wt | Yes | 21 | No | Yes | ileocec res |
| 11a | MZ | CCD | 1986 | F | snp 8 | No | 4 | No | No | No |
| 11b | MZ | H | 1986 | F | snp 8 | No | na | No | No | No |
| 12a | MZ | H | 1953 | M | wt | No | na | No | Yes | No |
| 12b | MZ | CCD | 1953 | M | wt | No | 33 | No | Yes | ileocec res |
| 13a | MZ | CCD | 1943 | F | wt | No | 18 | No | Yes | segm colonic rers |
| 13b | MZ | H | 1943 | F | wt | No | na | No | No | No |
| 14a | MZ | H | 1936 | M | wt | No | na | No | Yes | No |
| 14b | MZ | CCD | 1936 | M | wt | No | 11 | Yes | No | No |
| 15a | MZ | ICD | 1953 | M | snp 8 | No | 31 | No | No | ileal res |
| 15b | MZ | ICD | 1953 | M | snp 8 | No | 31 | No | No | ileocec res |
| 16a | MZ | ICD | 1954 | F | wt | No | 33 | No | Yes | ileo res + right hemi |
| 16b | MZ | H | 1954 | F | wt | No | na | No | No | No |
| 17a | MZ | CCD | 1976 | M | wt | No | 11 | No | Yes | No |
| 17b | MZ | CCD | 1976 | M | wt | No | 11 | Yes | Yes | No |
| 18a | MZ | ICD | 1953 | M | wt | No | 34 | No | No | ileal res + right hemi |
| 18b | MZ | H | 1953 | M | wt | No | na | No | No | No |
| Abbreviations: ileal res, ileal resection; right hemi, right sided hemicolectomy; ileocec res, ileocecal resection; segm colonic res, segmental colonic resection; na, not applicable; nd, no data; wt, wildtype; CCD, colonic Crohn’s disease; ICD, ileal Crohn’s disease; H, healthy co-twin: HH, healthy twin pair. NSAID, use of non-steroidal anti-inflammatory drugs within the preceding 12 months. Gastroenteritis within the last 3 months. Same number for twin sets, followed by a small letter “a” or “b” for each individual in a twin pair. (adapted from *1*) | | | | | | | | | | |
