# Supplementary material for: Metabolomics Reveals Metabolic Biomarkers of Crohn's Disease
Source: PLoS One. 2009 Jul 28;4(7):e6386. doi: 10.1371/journal.pone.0006386 (PMC2713417; doi:10.1371/journal.pone.0006386)
Supplement: Table S2 — Masses defined through the analysis presented in 5a) and assigned in MassTRIX (0.11 MB DOC) [file pone.0006386.s003.doc]

**Table S2:** Masses defined through the analysis presented in 5a) and assigned in MassTRIX.

**Bact Corr. ICD Raw mass neutral mass error [ppm] KEGG formula Name and Description**

Bacteroides ovatus (BO) (+) 514.284448 515.2916738 0.10 C26H45NO7S Taurocholic acid (Bile)

Bacteroides ovatus (BO) (+) 508.32799 509.3352528 0.03 C28H47NO7 Narbomycin (macrolyde)

Bacteroides ovatus (BO) (+) 407.2802529 408.2875744 -0.11 C24H40O5 Cholic acid (C24 bile acids)

Bacteroides ovatus (BO) (+) 179.0349986 180.0422587 0.09 C9H8O4 3-(4-Hydroxyphenyl)pyruvate/aspirin

Bacteroides ovatus (BO) (+) 328.1190667 329.1263227 0.06 C18H19NO5 1-O-Acetyllycorine (plant alkaloid)

Bacteroides vulgatus (BV) (+) 449.3271891 450.3345246 -0.13 C27H46O5 Coprocholic acid (C27 bile acids)

Bacteroides vulgatus (BV) (+) 285.243554 286.2507949 0.12 C17H34O3 methoxy or hydroxy (fatty acid)

Bacteroides vulgatus (BV) (+) 299.2591449 300.266445 -0.08 C18H36O3 hydroxy stearic acid (fatty acid)

Bacteroides vulgatus (BV) (+) 329.2333294 330.2406242 -0.06 C18H34O5 trihydroxy octadecenoic acid (fatty acid)

Bacteroides vulgatus (BV) (+) 243.1714022 244.1786926 -0.06 C12H24N2O3 Leucyl-leucine

Bacteroides vulgatus (BV) (+) 329.2487 330.2558803 0.29 C22H34O2 Docosapentaenoic acid (fatty acid)

Bacteroides vulgatus (BV) (+) 217.1194 218.1266571 0.09 C9H18N2O4 N2-(D-1-Carboxyethyl)-L-lysine

E. coli (EC) (+) 280.1190688 281.1263227 0.08 C14H19NO5 Harzianopyridone (prod. by fungus)

E. coli (EC) (+) 508.32799 509.3352528 0.03 C28H47NO7 Narbomycin (antibiotic)

E. coli (EC) (+) 531.114415 532.1216908 0.00 C25H24O13 Malonylglycitin (isoflavone from soy)

Faecalibacterium (FB) (-) 373.2747667 374.2820951 -0.14 C24H38O3 3-Oxo-5beta-cholanate (C24 bile acid)

Faecalibacterium (FB) (-) 455.0971755 456.1046149 -0.36 C17H21N4O9P FMN

Faecalibacterium (FB) (-) 456.177692 457.1849002 0.15 C23H27N3O7 Minocycline

Faecalibacterium (FB) (-) 447.3115566 448.3188745 -0.09 C27H44O5 2-Deoxyecdysone (C27 bile acids)

Faecalibacterium (FB) (-) 150.0560175 151.0633285 -0.23 C8H9NO2 Acetaminophen or (Z)-4- Hydroxyphenylacetaldehyde-oxime

Bacteroides uniformis (BU) (-) 337.202045 338.2093241 -0.01 C19H30O5 Shiromodiol diacetate

Bacteroides uniformis (BU) (-) 315.1237634 316.1310737 -0.11 C18H20O5 Sorgolactone

Bacteroides uniformis (BU) (-) 398.1245683 399.131802 0.11 C21H21NO7 Narcotoline

Bacteroides uniformis (BU) (-) 297.1132029 298.1205091 -0.10 C18H18O4 Enterolactone

Bacteroides uniformis (BU) (-) 403.1510233 404.1583511 -0.13 C20H24N2O7 Myxochelin A
